# Synthesis and structure of 6-bromo-2-(di­eth­oxymeth­yl)-2-hy­droxy-3-phenyl-2,3-di­hydro-1*H*-imidazo[1,2-*a*]pyridin-4-ium chloride aceto­nitrile monosolvate

**DOI:** 10.1107/S2056989025005778

**Published:** 2025-07-04

**Authors:** Firudin I. Guseinov, Aida I. Samigullina, Tuncer Hökelek, Sahil Z. Hamidov, Jamal Lasri, Khudayar I. Hasanov, Tahir A. Javadzade, Alebel N. Belay

**Affiliations:** a Kosygin State University of Russia, 117997 Moscow, Russian Federation; b N. D. Zelinsky Institute of Organic Chemistry, Russian Academy of Sciences, 119991 Moscow, Russian Federation; c Hacettepe University, Department of Physics, 06800 Beytepe-Ankara, Türkiye; d Azerbaijan Technological University, Shah Ismayil Khatai Avenue 103, AZ2011 Ganja, Azerbaijan; e Department of Chemistry Rabigh College of Science and Arts King Abdulaziz University,Jeddah 21589 Saudi Arabia; f Azerbaijan Medical University, Scientific Research Centre (SCR), A. Kasumzade St. 14, AZ1022 Baku, Azerbaijan; g Department of Chemistry and Chemical Engineering, Khazar University, Mahsati St. 41, AZ1096 Baku, Azerbaijan; h Department of Chemistry, Bahir Dar University, PO Box 79, Bahir Dar, Ethiopia; University of Aberdeen, United Kingdom

**Keywords:** crystal structure, non-covalent inter­actions, hydrogen bond, imidazole

## Abstract

In the title solvated mol­ecular salt, C_18_H_22_BrN_2_O_3_^+^·Cl^−^·CH_3_CN, the imidazole ring is in envelope conformation and the pyridine and phenyl rings are oriented at a dihedral angle of 72.52 (5)°. In the crystal, O—H⋯Cl and N—H⋯Cl hydrogen bonds link the cations and anions into centrosymmetric tetra­mers enclosing *R*^2^_4_(12) loops. Short Br⋯Cl [3.2313 (4) Å] and O⋯Cl [3.0490 (10) Å] contacts are observed. A Hirshfeld surface analysis of the structure indicates that the most important contributions for the crystal packing are from H⋯H (52.4%), H⋯C/C⋯H (12.1%), H⋯Br/Br⋯H (11.0%) and H⋯Cl/Cl⋯H (10.2%) inter­actions.

## Abstract 2

In the crystal of the title solvate, O—H⋯Cl and N—H⋯Cl hydrogen bonds link the cations and anions into centrosymmetric tetra­mers.

## 1. Chemical context

Nitro­gen-containing heterocycles, cyclic mol­ecules with one or more nitro­gen atoms in the cyclic scaffold, are ubiquitous in pharmaceutical drugs, agrochemicals, metalloenzymes and biologically active natural products (Li *et al.*, 2023 ▸). The synthetic chemistry of N-heterocycles is not limited to organic chemistry (Guseinov *et al.*, 2017 ▸, 2020 ▸, 2024 ▸), they have been well explored in the spectrophotometric determination of metal ions (Alieva *et al.*, 2008 ▸), synthesis of cyclic carbonates from cyclo­addition of CO_2_ with epoxides (Aliyeva *et al.*, 2024 ▸), crystal engineering (Naghiyev *et al.*, 2023 ▸) and catalysis (Kerimli *et al.*, 2021 ▸). N-heterocycles have many advantages, such as easy modification and functionalization (Khalilov *et al.*, 2021 ▸), immobilization on solid materials through supra­molecular inter­actions (Mammadov *et al.*, 2023 ▸) and crystal growth and design (Hajiyeva *et al.*, 2024 ▸). As part of our work in this area, we now describe the synthesis and structure of the title solvated mol­ecular salt, C_18_H_22_BrN_2_O_3_^+^·Cl ^−^·C_2_H_3_N (**1**).
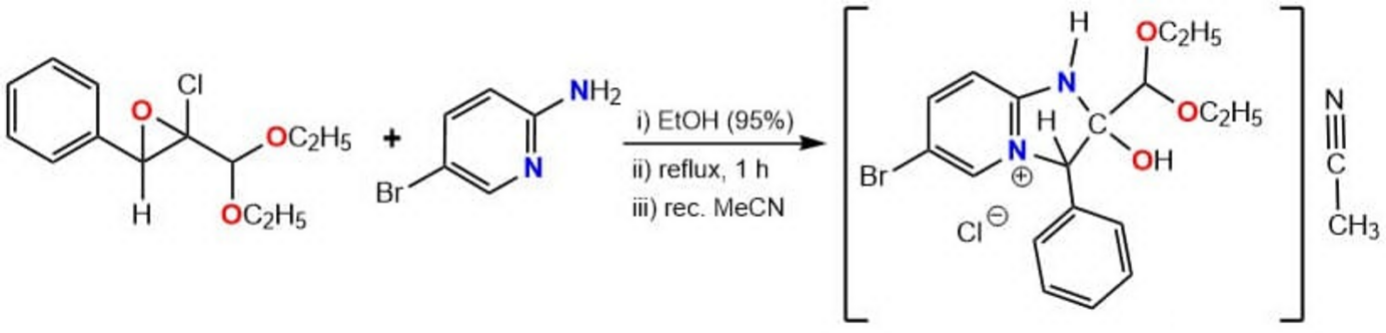


## 2. Structural commentary

**Figure 1:**
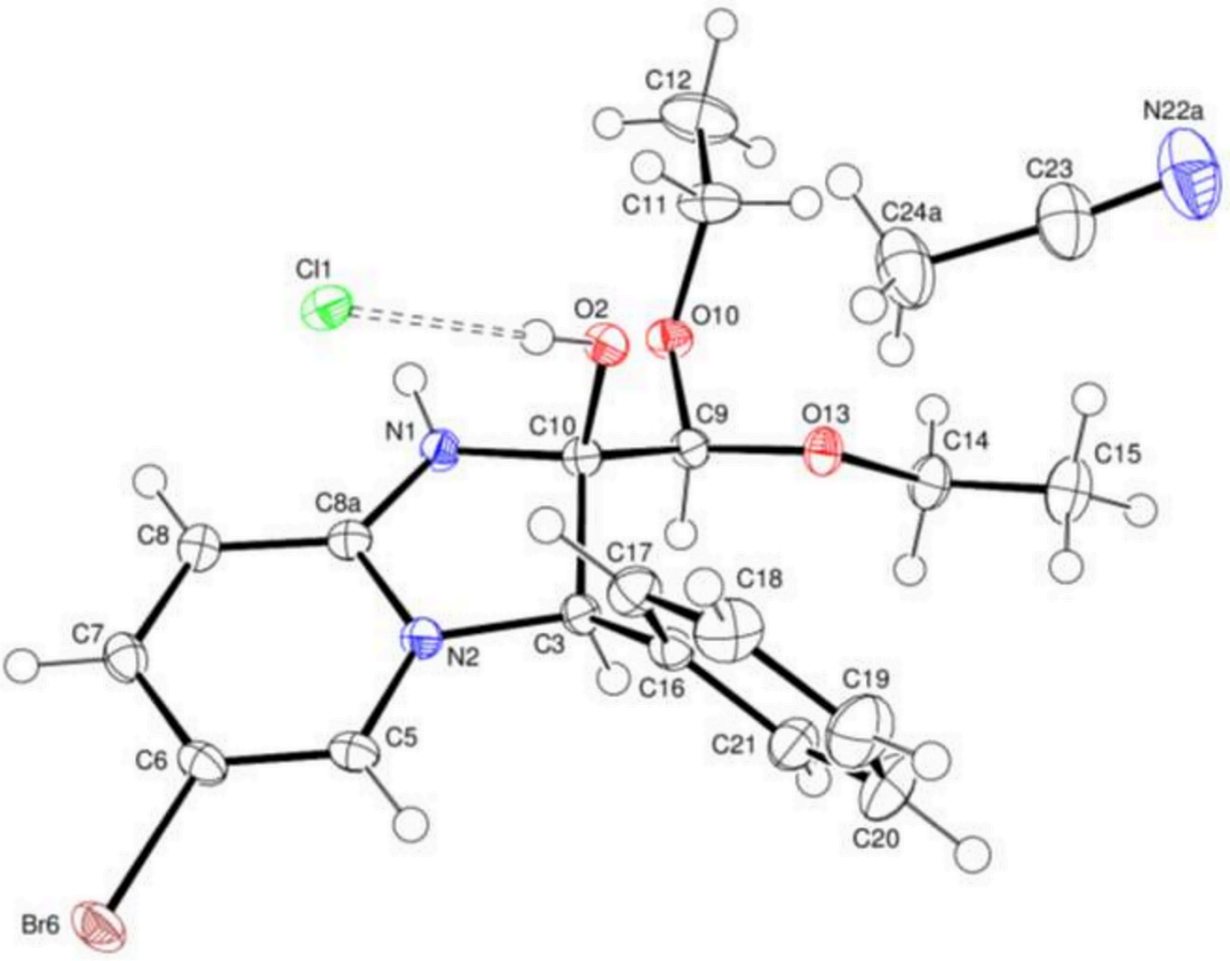
The asymmetric unit of the title compound with 50% probability ellipsoids. Only one part of the disordered atoms is shown for clarity. The O—H⋯Cl hydrogen bond is shown as a dashed line.

The mol­ecular structure of (**1**) is illustrated in Fig. 1 ▸. In the cation, the five-membered imidazole N1/N2/C3/C8*A*/C10 ring is non-planar due to the substituents bonded to atoms C3 and C10. It adopts an envelope conformation with puckering parameter φ = 137.38 (5)° where atom C10 is at the flap position and is displaced by −0.4138 (13) Å from the best least-squares plane of the other four atoms. The pyridine N2/C5–C8/C8a and phenyl C16–C21 rings are oriented at a dihedral angle of 72.52 (5)°. Atom Br6 is displaced by −0.0523 (1) Å from the best least-squares plane of the pyridine ring. The pendant C12 eth­oxy­methyl group has a *gauche*–*anti* conformation as indicated by the following torsion angles: C10—C9—O10—C11 = 75.76 (15)°; C9—O10—C11—C12 = 170.67 (13)°. Conversely, the C15 chain is *anti*–*anti*: C10—C9—O13—C14 = 163.79 (11)°; C9—O13—C14—C15 = −176.75 (12)°. Atom N1 of the imidazole ring is *anti* to O13 [N1—C10—C9—O13 = 178.28 (10)°] and *gauche* to O10 [N1—C10—C9—O10 = 51.69 (14)°]. Atoms C3 and C10 of the cation are stereogenic centres: in the arbitrarily chosen asymmetric unit, they both have *R* configurations, but crystal symmetry generates a racemic mixture.

## 3. Supra­molecular features

**Figure 2:**
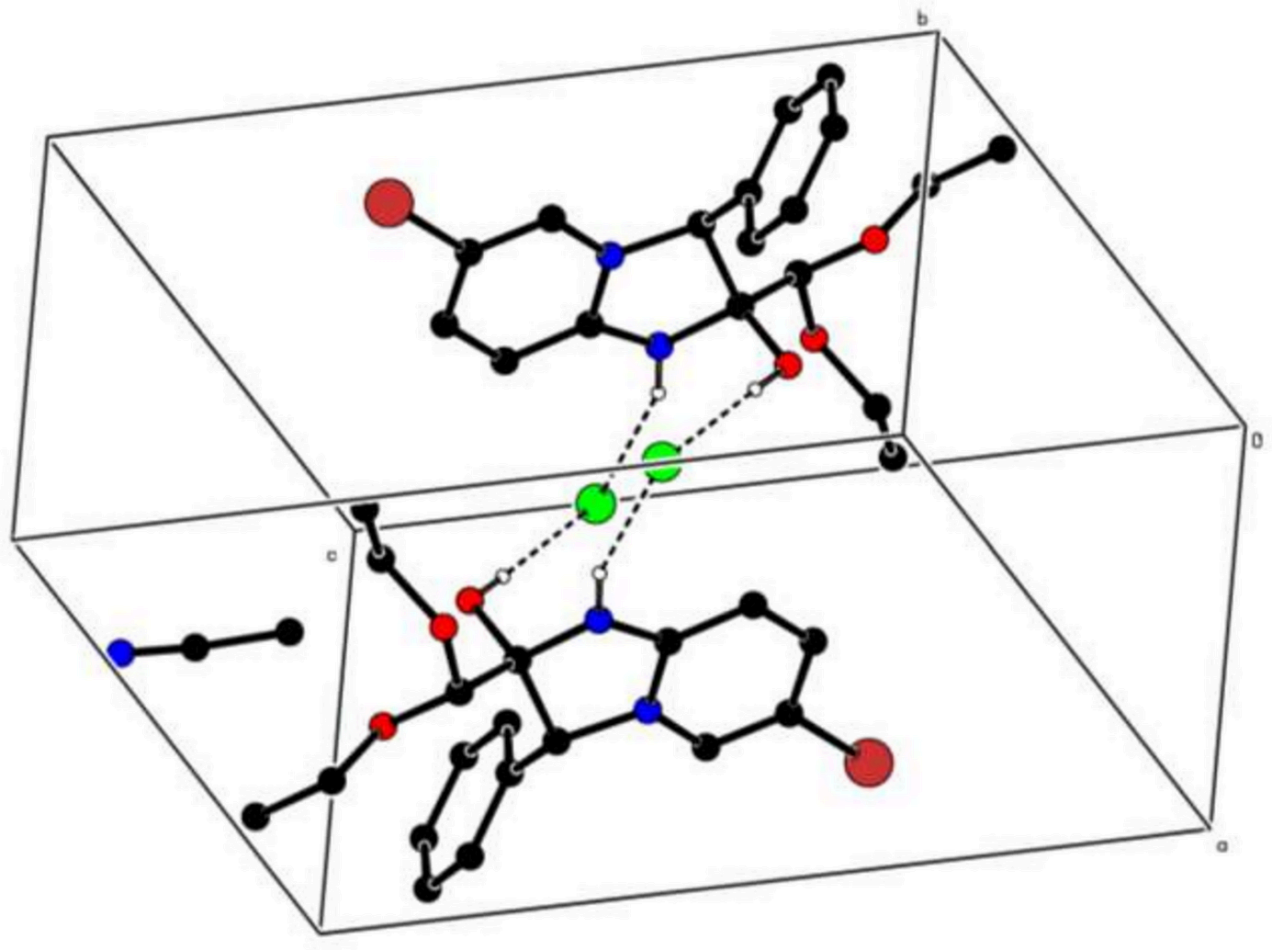
A partial packing diagram with O—H⋯Cl and N—H⋯Cl hydrogen bonds shown as dashed lines. The other hydrogen atoms have been omitted for clarity.

In the crystal, the cation and the anion are linked by a strong O—H⋯Cl hydrogen bond (Table 1 ▸). The chloride ion also accepts an N—H⋯Cl hydrogen bond from another cation, which generates centrosymmetric tetra­mers (two cations, two anions) enclosing 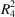
(12) loops (Fig. 2 ▸). Various weak C—H⋯N, C—H⋯O and C—H⋯Cl hydrogen bonds are also observed (Table 1 ▸). Short Br6⋯Cl1 [3.2313 (4) Å, compared to a van der Waals separation of about 3.60 Å] and O2⋯Cl1 [3.0490 (10), 3.27 Å] contacts occur.

## 4. Hirshfeld surface analysis

**Figure 3:**
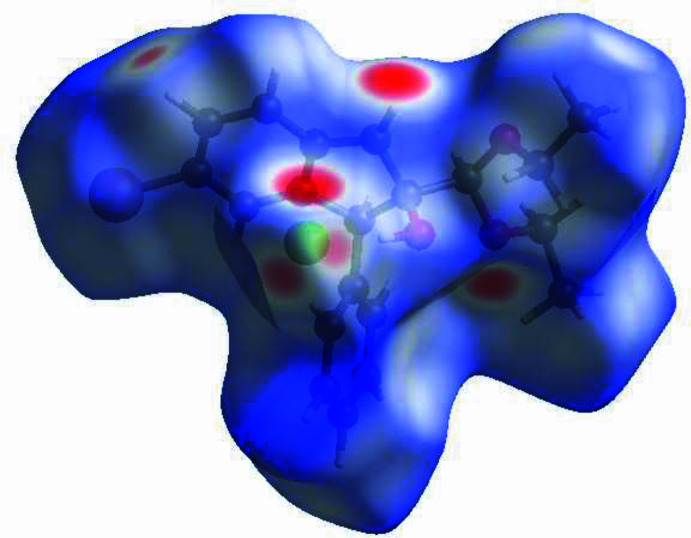
View of the three-dimensional Hirshfeld surface of the title compound plotted over *d*_norm_.

**Figure 4:**
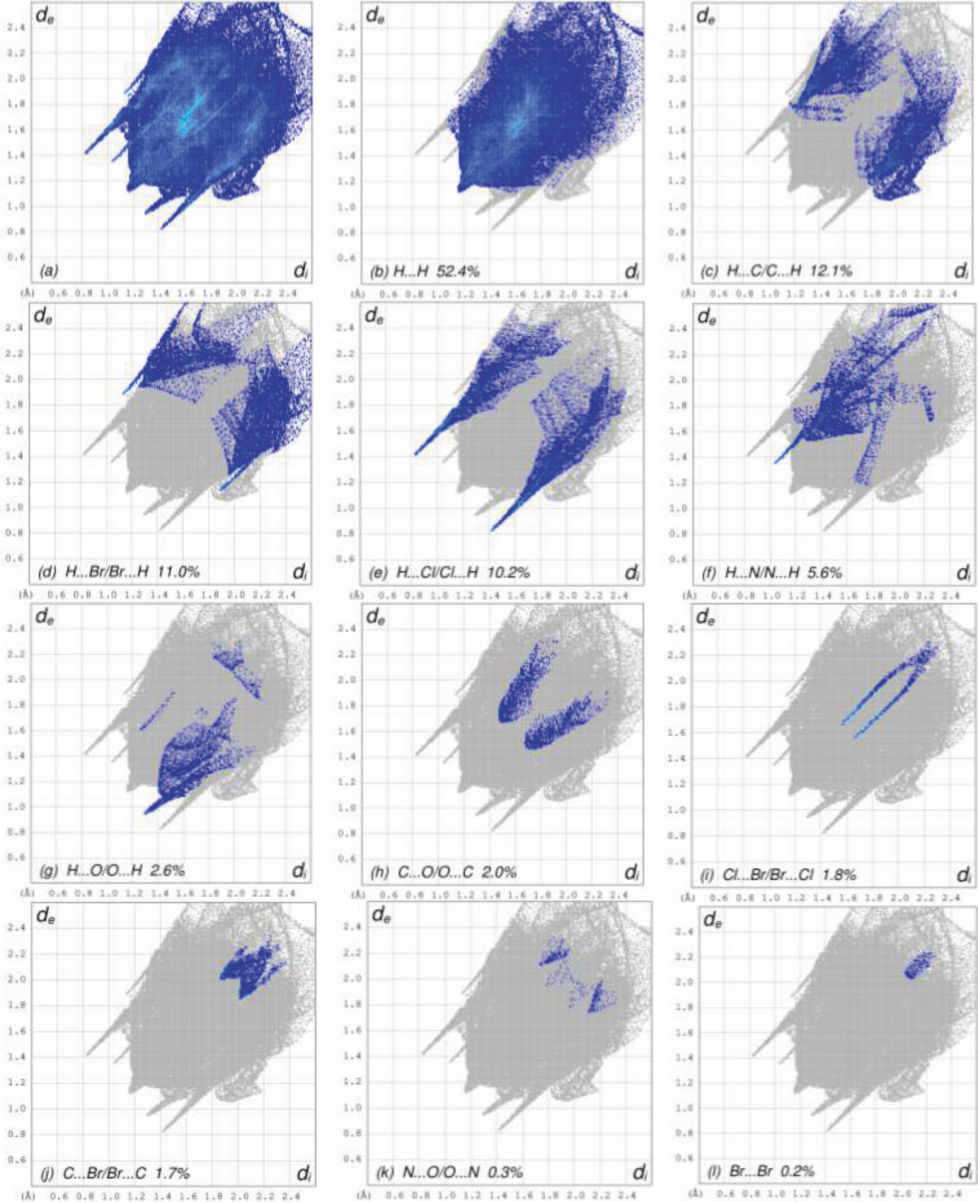
The two-dimensional fingerprint plots for the title compound, showing (*a*) all inter­actions and (*b*)–(*n*) different contact types. The *d*_i_ and *d*_e_ values are the closest inter­nal and external distances (in Å) from given points on the Hirshfeld surface. Both disordered components of the acetonitrile molecule were omitted.

To visualize the inter­molecular inter­actions in the crystal a Hirshfeld surface (HS) analysis was carried out using *Crystal Explorer 17.5* (Spackman *et al.*, 2021 ▸) following the protocol of Tan *et al.* (2019 ▸) after removal of the disordered aceto­nitrile solvent mol­ecule. In the surface plotted over *d*_norm_ (Fig. 3 ▸), the contact distances equal, shorter and longer with respect to the sum of van der Waals radii are shown by the white, red and blue colours, respectively, where the bright-red spots correspond to the respective donors and/or acceptors. The overall two-dimensional fingerprint plot, Fig. 4 ▸*a*, and those delineated into the different contact types are illustrated in Fig. 4 ▸*b–n*, together with their relative contributions to the HS.The most important contributors to the surface are H⋯H (52.4%) H⋯C/C⋯H (12.1%), H⋯Br/Br⋯H (11.0%) and H⋯Cl/Cl⋯H (10.2%) contacts. The remaining contacts have a very low density of points.

## 5. Synthesis and crystallization

A solution of 2-chloro-2-(di­eth­oxy­meth­yl)-3-phenyl­oxirane (128 mg, 0.5 mmol) and 5-bromo­pyridin-2-amine (82 mg, 0.5 mmol) in 20 ml of ethanol (95%) was boiled for 1 h. The solvent was distilled off *in vacuo* and the remaining powder was recrystallized from aceto­nitrile solution. Yield: 166 mg (79%), m.p. 485–487 K. Analysis calculated (%) for C_20_H_25_BrClN_3_O_3_: C 51.02, H 5.35, N 8.93; found C 51.00, H 5.32, N 8.91. ^1^H NMR (300 MHz, DMSO-*d*_6_): 1.22 (6H), 2.07 (3H), 3.63–3.94 (4H), 4.77 (1H), 6.43 (1H), 6.89 (OH), 7.43–7.93 (8H), 8.25 (NH), ^13^C NMR (200 MHz, DMSO-d^6^): 1.03, 15.55, 63.56, 76.28, 101.78, 104.25, 111.25, 117.90, 125.58, 126.77, 128.88, 129.25, 143.89, 147.25, 151.26, 160.99.

## 6. Refinement

Crystal data, data collection and structure refinement details are summarized in Table 2 ▸. The OH and NH hydrogen atoms were located in a difference-Fourier map and refined isotropically. The C-bound hydrogen-atom positions were calculated geometrically at distances of 0.95–1.00 Å depending on hybridization and refined using a riding model by applying the constraints of *U*_iso_ = 1.2*U*_eq_(C) or 1.5*U*_eq_(methyl C). Atoms N22 and C24 and its attached H atoms of the aceto­nitrile solvent mol­ecule are disordered over two adjacent orientations, and they were refined with an occupancy ratio of 0.443 (19):0.557 (19).

## Supplementary Material

Crystal structure: contains datablock(s) I. DOI: 10.1107/S2056989025005778/hb8130sup1.cif

Structure factors: contains datablock(s) I. DOI: 10.1107/S2056989025005778/hb8130Isup2.hkl

Supporting information file. DOI: 10.1107/S2056989025005778/hb8130Isup3.cml

CCDC reference: 2467703

Additional supporting information:  crystallographic information; 3D view; checkCIF report

## Figures and Tables

**Table 1:** Hydrogen-bond geometry (Å, °)

*D*—H⋯*A*	*D*—H	H⋯*A*	*D*⋯*A*	*D*—H⋯*A*
O2—H2⋯Cl1	0.80 (2)	2.25 (2)	3.0489 (10)	177 (2)
N1—H1⋯Cl1^i^	0.86 (2)	2.37 (2)	3.1829 (12)	157.0 (17)
C7—H7⋯N22*A*^ii^	0.95	2.51	3.410 (15)	157
C24*A*—H24*F*⋯O13	0.98	2.34	3.260 (12)	156
C3—H3⋯Cl1^iii^	1.00	2.62	3.6125 (13)	171
C9—H9⋯Cl1^iii^	1.00	2.72	3.6643 (14)	157
C17—H17⋯Cl1	0.95	2.70	3.6259 (15)	165

Symmetry codes: (i) 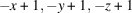; (ii) 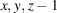; (iii) 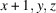.

**Table 2:** Experimental details

Crystal data	
Chemical formula	C_18_H_22_BrN_2_O_3_^+^·Cl^−^·C_2_H_3_N
*M*_r_	470.79
Crystal system, space group	Triclinic, *P* 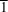
Temperature (K)	100
*a*, *b*, *c* (Å)	7.59344 (7), 11.07365 (13), 13.73164 (16)
α, β, γ (°)	75.4564 (10), 85.3656 (8), 88.4658 (8)
*V* (Å^3^)	1113.98 (2)
*Z*	2
Radiation type	Cu *K*α
μ (mm^−1^)	3.82
Crystal size (mm)	0.29 × 0.22 × 0.18

Data collection	
Diffractometer	XtaLAB Synergy, Dualflex, HyPix
Absorption correction	Gaussian (*CrysAlis PRO*; Rigaku OD, 2023 ▸)
*T*_min_, *T*_max_	0.345, 1.000
No. of measured, independent and observed [*I* > 2σ(*I*)] reflections	28449, 4661, 4621
*R*_int_	0.025
(sin θ/λ)_max_ (Å^−1^)	0.632

Refinement	
*R*[*F*^2^ > 2σ(*F*^2^)], *wR*(*F*^2^), *S*	0.023, 0.058, 1.07
No. of reflections	4661
No. of parameters	273
No. of restraints	2
H-atom treatment	H atoms treated by a mixture of independent and constrained refinement
Δρ_max_, Δρ_min_ (e Å^−3^)	0.34, −0.36

Computer programs: *CrysAlis PRO* (Rigaku OD, 2023 ▸), *SHELXT2014/5* (Sheldrick, 2015*a* ▸), *SHELXL2018/3* (Sheldrick, 2015*b* ▸) and *OLEX2* (Dolomanov *et al.*, 2009 ▸).

## supplementary crystallographic information

### 6-Bromo-2-(diethoxymethyl)-2-hydroxy-3-phenyl-2,3-dihydro-1H-imidazo[1,2-a]pyridin-4-ium chloride acetonitrile monosolvate Crystal data

**Table:**

C_18_H_22_BrN_2_O_3_^+^·Cl^−^·C_2_H_3_N	*Z* = 2
*M**_r_* = 470.79	*F*(000) = 484
Triclinic, *P*1	*D*_x_ = 1.404 Mg m^−^^3^
*a* = 7.59344 (7) Å	Cu *K*α radiation, λ = 1.54184 Å
*b* = 11.07365 (13) Å	Cell parameters from 22359 reflections
*c* = 13.73164 (16) Å	θ = 3.3–76.8°
α = 75.4564 (10)°	µ = 3.82 mm^−^^1^
β = 85.3656 (8)°	*T* = 100 K
γ = 88.4658 (8)°	Prism, colorless
*V* = 1113.98 (2) Å^3^	0.29 × 0.22 × 0.18 mm

### 6-Bromo-2-(diethoxymethyl)-2-hydroxy-3-phenyl-2,3-dihydro-1H-imidazo[1,2-a]pyridin-4-ium chloride acetonitrile monosolvate Data collection

**Table:**

XtaLAB Synergy, Dualflex, HyPix diffractometer	4661 independent reflections
Radiation source: micro-focus sealed X-ray tube, PhotonJet (Cu) X-ray Source	4621 reflections with *I* > 2σ(*I*)
Mirror monochromator	*R*_int_ = 0.025
Detector resolution: 10.0000 pixels mm^-1^	θ_max_ = 77.0°, θ_min_ = 3.3°
ω scans	*h* = −9→8
Absorption correction: gaussian (CrysAlisPro; Rigaku OD, 2023)	*k* = −13→13
*T*_min_ = 0.345, *T*_max_ = 1.000	*l* = −17→17
28449 measured reflections	

### 6-Bromo-2-(diethoxymethyl)-2-hydroxy-3-phenyl-2,3-dihydro-1H-imidazo[1,2-a]pyridin-4-ium chloride acetonitrile monosolvate Refinement

**Table:**

Refinement on *F*^2^	Secondary atom site location: difference Fourier map
Least-squares matrix: full	Hydrogen site location: mixed
*R*[*F*^2^ > 2σ(*F*^2^)] = 0.023	H atoms treated by a mixture of independent and constrained refinement
*wR*(*F*^2^) = 0.058	*w* = 1/[σ^2^(*F*_o_^2^) + (0.0263*P*)^2^ + 0.6353*P*] where *P* = (*F*_o_^2^ + 2*F*_c_^2^)/3
*S* = 1.07	(Δ/σ)_max_ = 0.002
4661 reflections	Δρ_max_ = 0.34 e Å^−^^3^
273 parameters	Δρ_min_ = −0.36 e Å^−^^3^
2 restraints	Extinction correction: *SHELXL2018/3* (Sheldrick, 2015b), Fc^*^=kFc[1+0.001xFc^2^λ^3^/sin(2θ)]^-1/4^
Primary atom site location: dual	Extinction coefficient: 0.00204 (13)

### 6-Bromo-2-(diethoxymethyl)-2-hydroxy-3-phenyl-2,3-dihydro-1H-imidazo[1,2-a]pyridin-4-ium chloride acetonitrile monosolvate Special details

**Table:**

Geometry. All esds (except the esd in the dihedral angle between two l.s. planes) are estimated using the full covariance matrix. The cell esds are taken into account individually in the estimation of esds in distances, angles and torsion angles; correlations between esds in cell parameters are only used when they are defined by crystal symmetry. An approximate (isotropic) treatment of cell esds is used for estimating esds involving l.s. planes.

### 6-Bromo-2-(diethoxymethyl)-2-hydroxy-3-phenyl-2,3-dihydro-1H-imidazo[1,2-a]pyridin-4-ium chloride acetonitrile monosolvate Fractional atomic coordinates and isotropic or equivalent isotropic displacement parameters (Å^2^)

**Table:**

	*x*	*y*	*z*	*U*_iso_*/*U*_eq_	Occ. (<1)
Br6	0.71051 (2)	−0.02116 (2)	0.40214 (2)	0.02342 (6)	
O2	0.63388 (13)	0.39419 (9)	0.70984 (7)	0.01627 (19)	
H2	0.555 (3)	0.369 (2)	0.6849 (16)	0.034 (5)*	
O10	0.90770 (13)	0.59295 (9)	0.65996 (7)	0.01821 (19)	
O13	0.94075 (12)	0.41038 (9)	0.78971 (7)	0.01768 (19)	
N1	0.77063 (15)	0.44100 (11)	0.54370 (8)	0.0156 (2)	
H1	0.735 (2)	0.5163 (19)	0.5181 (14)	0.023 (4)*	
N2	0.79268 (14)	0.23811 (10)	0.55778 (8)	0.0148 (2)	
C3	0.86257 (17)	0.25545 (12)	0.65138 (10)	0.0149 (2)	
H3	0.994348	0.259107	0.640632	0.018*	
C5	0.78508 (17)	0.12883 (13)	0.53030 (10)	0.0174 (3)	
H5	0.818893	0.052418	0.574201	0.021*	
C6	0.72782 (18)	0.13148 (13)	0.43832 (11)	0.0184 (3)	
C7	0.67733 (18)	0.24541 (14)	0.37389 (10)	0.0191 (3)	
H7	0.637428	0.246615	0.309840	0.023*	
C8	0.68567 (17)	0.35439 (13)	0.40341 (10)	0.0177 (3)	
H8	0.650962	0.431486	0.360816	0.021*	
C8A	0.74689 (17)	0.34921 (12)	0.49836 (10)	0.0150 (2)	
C9	0.93386 (17)	0.46309 (12)	0.68605 (10)	0.0153 (2)	
H9	1.049840	0.445590	0.652129	0.018*	
C10	0.79212 (17)	0.39016 (12)	0.65150 (10)	0.0143 (2)	
C11	0.7717 (2)	0.64392 (14)	0.71812 (13)	0.0272 (3)	
H11A	0.785968	0.611957	0.791164	0.033*	
H11B	0.653457	0.619906	0.704179	0.033*	
C12	0.7906 (3)	0.78235 (16)	0.68768 (17)	0.0405 (4)	
H12A	0.781124	0.812412	0.614806	0.061*	
H12B	0.906170	0.805053	0.704541	0.061*	
H12C	0.697028	0.820516	0.723683	0.061*	
C14	1.09708 (19)	0.44236 (15)	0.83005 (11)	0.0238 (3)	
H14A	1.100746	0.533414	0.823262	0.029*	
H14B	1.204216	0.417686	0.793453	0.029*	
C15	1.0887 (2)	0.37254 (18)	0.94002 (13)	0.0331 (4)	
H15A	1.085856	0.282624	0.945606	0.050*	
H15B	0.981776	0.397466	0.975210	0.050*	
H15C	1.193030	0.392261	0.970583	0.050*	
C16	0.81484 (18)	0.15009 (12)	0.74240 (10)	0.0170 (3)	
C17	0.64230 (19)	0.10643 (13)	0.76702 (11)	0.0204 (3)	
H17	0.550314	0.143950	0.726404	0.024*	
C18	0.6051 (2)	0.00806 (15)	0.85096 (12)	0.0287 (3)	
H18	0.487323	−0.021077	0.868097	0.034*	
C19	0.7394 (3)	−0.04782 (16)	0.90987 (13)	0.0360 (4)	
H19	0.713307	−0.114718	0.967518	0.043*	
C20	0.9118 (2)	−0.00599 (16)	0.88460 (13)	0.0338 (4)	
H20	1.003978	−0.045215	0.924294	0.041*	
C21	0.9496 (2)	0.09330 (14)	0.80120 (11)	0.0239 (3)	
H21	1.067421	0.122388	0.784344	0.029*	
Cl1	0.33366 (4)	0.30609 (3)	0.61057 (2)	0.01733 (8)	
N22	0.602 (7)	0.357 (3)	1.1158 (12)	0.046 (3)	0.443 (19)
N22A	0.594 (5)	0.331 (2)	1.1255 (9)	0.046 (3)	0.557 (19)
C24	0.611 (2)	0.2884 (16)	0.9482 (9)	0.0410 (19)	0.443 (19)
H24A	0.686904	0.347333	0.898044	0.061*	0.443 (19)
H24B	0.493588	0.288120	0.923808	0.061*	0.443 (19)
H24C	0.663150	0.204517	0.959064	0.061*	0.443 (19)
C24A	0.5848 (18)	0.3253 (13)	0.9381 (7)	0.0410 (19)	0.557 (19)
H24D	0.517870	0.398668	0.904347	0.061*	0.557 (19)
H24E	0.524540	0.249411	0.934754	0.061*	0.557 (19)
H24F	0.703877	0.327211	0.904437	0.061*	0.557 (19)
C23	0.5968 (2)	0.32604 (18)	1.04328 (13)	0.0337 (4)	

### 6-Bromo-2-(diethoxymethyl)-2-hydroxy-3-phenyl-2,3-dihydro-1H-imidazo[1,2-a]pyridin-4-ium chloride acetonitrile monosolvate Atomic displacement parameters (Å^2^)

**Table:**

	*U*^11^	*U*^22^	*U*^33^	*U*^12^	*U*^13^	*U*^23^
Br6	0.02516 (9)	0.02146 (9)	0.02878 (9)	−0.00157 (6)	−0.00279 (6)	−0.01548 (6)
O2	0.0129 (4)	0.0182 (5)	0.0193 (5)	−0.0019 (3)	−0.0004 (4)	−0.0077 (4)
O10	0.0188 (5)	0.0135 (4)	0.0231 (5)	−0.0011 (3)	−0.0015 (4)	−0.0059 (4)
O13	0.0174 (5)	0.0188 (5)	0.0175 (5)	−0.0031 (4)	−0.0041 (4)	−0.0047 (4)
N1	0.0177 (5)	0.0134 (5)	0.0162 (5)	−0.0005 (4)	−0.0033 (4)	−0.0038 (4)
N2	0.0138 (5)	0.0148 (5)	0.0163 (5)	−0.0006 (4)	−0.0013 (4)	−0.0045 (4)
C3	0.0150 (6)	0.0143 (6)	0.0164 (6)	−0.0010 (5)	−0.0025 (5)	−0.0054 (5)
C5	0.0167 (6)	0.0145 (6)	0.0220 (7)	−0.0004 (5)	0.0007 (5)	−0.0072 (5)
C6	0.0169 (6)	0.0184 (6)	0.0224 (7)	−0.0025 (5)	0.0009 (5)	−0.0106 (5)
C7	0.0169 (6)	0.0241 (7)	0.0175 (6)	−0.0030 (5)	−0.0001 (5)	−0.0075 (5)
C8	0.0168 (6)	0.0192 (6)	0.0164 (6)	−0.0018 (5)	−0.0013 (5)	−0.0030 (5)
C8A	0.0119 (6)	0.0148 (6)	0.0178 (6)	−0.0015 (4)	0.0008 (5)	−0.0038 (5)
C9	0.0152 (6)	0.0142 (6)	0.0174 (6)	−0.0012 (5)	−0.0022 (5)	−0.0050 (5)
C10	0.0138 (6)	0.0139 (6)	0.0154 (6)	−0.0010 (4)	−0.0010 (5)	−0.0038 (5)
C11	0.0204 (7)	0.0205 (7)	0.0425 (9)	0.0018 (5)	0.0010 (6)	−0.0125 (6)
C12	0.0440 (10)	0.0218 (8)	0.0572 (12)	−0.0017 (7)	0.0097 (9)	−0.0166 (8)
C14	0.0205 (7)	0.0283 (7)	0.0244 (7)	−0.0030 (6)	−0.0086 (6)	−0.0075 (6)
C15	0.0329 (9)	0.0399 (9)	0.0268 (8)	−0.0021 (7)	−0.0124 (7)	−0.0056 (7)
C16	0.0214 (7)	0.0136 (6)	0.0168 (6)	0.0009 (5)	−0.0020 (5)	−0.0049 (5)
C17	0.0218 (7)	0.0159 (6)	0.0228 (7)	−0.0004 (5)	−0.0025 (5)	−0.0034 (5)
C18	0.0308 (8)	0.0215 (7)	0.0293 (8)	−0.0035 (6)	0.0028 (6)	0.0005 (6)
C19	0.0466 (10)	0.0263 (8)	0.0271 (8)	0.0012 (7)	−0.0019 (7)	0.0076 (6)
C20	0.0390 (9)	0.0311 (9)	0.0271 (8)	0.0068 (7)	−0.0113 (7)	0.0027 (7)
C21	0.0241 (7)	0.0234 (7)	0.0241 (7)	0.0030 (6)	−0.0062 (6)	−0.0046 (6)
Cl1	0.01411 (14)	0.01354 (14)	0.02489 (16)	−0.00020 (10)	−0.00402 (11)	−0.00502 (11)
N22	0.046 (3)	0.065 (9)	0.026 (2)	−0.015 (7)	0.003 (3)	−0.010 (4)
N22A	0.046 (3)	0.065 (9)	0.026 (2)	−0.015 (7)	0.003 (3)	−0.010 (4)
C24	0.028 (4)	0.071 (7)	0.0266 (18)	−0.010 (3)	0.0026 (16)	−0.018 (3)
C24A	0.028 (4)	0.071 (7)	0.0266 (18)	−0.010 (3)	0.0026 (16)	−0.018 (3)
C23	0.0292 (8)	0.0458 (10)	0.0243 (8)	−0.0118 (7)	0.0026 (6)	−0.0056 (7)

### 6-Bromo-2-(diethoxymethyl)-2-hydroxy-3-phenyl-2,3-dihydro-1H-imidazo[1,2-a]pyridin-4-ium chloride acetonitrile monosolvate Geometric parameters (Å, º)

**Table:**

Br6—C6	1.8882 (13)	C12—H12B	0.9800
O2—H2	0.80 (2)	C12—H12C	0.9800
O2—C10	1.3954 (16)	C14—H14A	0.9900
O10—C9	1.4047 (16)	C14—H14B	0.9900
O10—C11	1.4438 (18)	C14—C15	1.511 (2)
O13—C9	1.4010 (16)	C15—H15A	0.9800
O13—C14	1.4383 (16)	C15—H15B	0.9800
N1—H1	0.86 (2)	C15—H15C	0.9800
N1—C8A	1.3413 (18)	C16—C17	1.395 (2)
N1—C10	1.4664 (16)	C16—C21	1.392 (2)
N2—C3	1.4867 (16)	C17—H17	0.9500
N2—C5	1.3590 (17)	C17—C18	1.389 (2)
N2—C8A	1.3487 (17)	C18—H18	0.9500
C3—H3	1.0000	C18—C19	1.387 (2)
C3—C10	1.5715 (18)	C19—H19	0.9500
C3—C16	1.5072 (18)	C19—C20	1.388 (3)
C5—H5	0.9500	C20—H20	0.9500
C5—C6	1.362 (2)	C20—C21	1.391 (2)
C6—C7	1.411 (2)	C21—H21	0.9500
C7—H7	0.9500	N22—C23	1.132 (12)
C7—C8	1.371 (2)	N22A—C23	1.141 (9)
C8—H8	0.9500	C24—H24A	0.9800
C8—C8A	1.4065 (19)	C24—H24B	0.9800
C9—H9	1.0000	C24—H24C	0.9800
C9—C10	1.5352 (17)	C24—C23	1.462 (11)
C11—H11A	0.9900	C24A—H24D	0.9800
C11—H11B	0.9900	C24A—H24E	0.9800
C11—C12	1.492 (2)	C24A—H24F	0.9800
C12—H12A	0.9800	C24A—C23	1.457 (8)
			
C10—O2—H2	109.4 (15)	C11—C12—H12B	109.5
C9—O10—C11	117.69 (11)	C11—C12—H12C	109.5
C9—O13—C14	113.71 (10)	H12A—C12—H12B	109.5
C8A—N1—H1	121.0 (12)	H12A—C12—H12C	109.5
C8A—N1—C10	110.85 (11)	H12B—C12—H12C	109.5
C10—N1—H1	123.7 (12)	O13—C14—H14A	110.3
C5—N2—C3	126.31 (11)	O13—C14—H14B	110.3
C8A—N2—C3	110.27 (11)	O13—C14—C15	106.88 (12)
C8A—N2—C5	123.28 (12)	H14A—C14—H14B	108.6
N2—C3—H3	108.0	C15—C14—H14A	110.3
N2—C3—C10	101.08 (10)	C15—C14—H14B	110.3
N2—C3—C16	112.88 (10)	C14—C15—H15A	109.5
C10—C3—H3	108.0	C14—C15—H15B	109.5
C16—C3—H3	108.0	C14—C15—H15C	109.5
C16—C3—C10	118.46 (11)	H15A—C15—H15B	109.5
N2—C5—H5	120.8	H15A—C15—H15C	109.5
N2—C5—C6	118.41 (13)	H15B—C15—H15C	109.5
C6—C5—H5	120.8	C17—C16—C3	122.00 (12)
C5—C6—Br6	118.27 (11)	C21—C16—C3	118.21 (13)
C5—C6—C7	120.32 (13)	C21—C16—C17	119.76 (13)
C7—C6—Br6	121.38 (10)	C16—C17—H17	120.0
C6—C7—H7	119.9	C18—C17—C16	119.93 (14)
C8—C7—C6	120.20 (13)	C18—C17—H17	120.0
C8—C7—H7	119.9	C17—C18—H18	119.9
C7—C8—H8	120.8	C19—C18—C17	120.21 (15)
C7—C8—C8A	118.33 (13)	C19—C18—H18	119.9
C8A—C8—H8	120.8	C18—C19—H19	120.0
N1—C8A—N2	110.41 (12)	C18—C19—C20	120.01 (15)
N1—C8A—C8	130.13 (13)	C20—C19—H19	120.0
N2—C8A—C8	119.46 (12)	C19—C20—H20	120.0
O10—C9—H9	107.4	C19—C20—C21	120.04 (15)
O10—C9—C10	113.93 (11)	C21—C20—H20	120.0
O13—C9—O10	114.38 (11)	C16—C21—H21	120.0
O13—C9—H9	107.4	C20—C21—C16	120.04 (15)
O13—C9—C10	105.91 (10)	C20—C21—H21	120.0
C10—C9—H9	107.4	H24A—C24—H24B	109.5
O2—C10—N1	111.60 (10)	H24A—C24—H24C	109.5
O2—C10—C3	115.02 (10)	H24B—C24—H24C	109.5
O2—C10—C9	109.37 (10)	C23—C24—H24A	109.5
N1—C10—C3	100.50 (10)	C23—C24—H24B	109.5
N1—C10—C9	110.23 (10)	C23—C24—H24C	109.5
C9—C10—C3	109.84 (10)	H24D—C24A—H24E	109.5
O10—C11—H11A	110.3	H24D—C24A—H24F	109.5
O10—C11—H11B	110.3	H24E—C24A—H24F	109.5
O10—C11—C12	107.15 (13)	C23—C24A—H24D	109.5
H11A—C11—H11B	108.5	C23—C24A—H24E	109.5
C12—C11—H11A	110.3	C23—C24A—H24F	109.5
C12—C11—H11B	110.3	N22—C23—C24	174 (3)
C11—C12—H12A	109.5	N22A—C23—C24A	175 (2)
			
Br6—C6—C7—C8	177.82 (10)	C7—C8—C8A—N2	−1.00 (19)
O10—C9—C10—O2	−71.36 (13)	C8A—N1—C10—O2	−97.51 (13)
O10—C9—C10—N1	51.69 (14)	C8A—N1—C10—C3	24.90 (13)
O10—C9—C10—C3	161.53 (10)	C8A—N1—C10—C9	140.75 (11)
O13—C9—C10—O2	55.23 (13)	C8A—N2—C3—C10	17.62 (13)
O13—C9—C10—N1	178.28 (10)	C8A—N2—C3—C16	145.19 (11)
O13—C9—C10—C3	−71.88 (13)	C8A—N2—C5—C6	−0.23 (19)
N2—C3—C10—O2	95.88 (12)	C9—O10—C11—C12	170.67 (13)
N2—C3—C10—N1	−24.09 (11)	C9—O13—C14—C15	−176.75 (12)
N2—C3—C10—C9	−140.24 (10)	C10—N1—C8A—N2	−15.17 (15)
N2—C3—C16—C17	−49.31 (17)	C10—N1—C8A—C8	165.20 (13)
N2—C3—C16—C21	128.79 (13)	C10—C3—C16—C17	68.46 (17)
N2—C5—C6—Br6	−178.08 (9)	C10—C3—C16—C21	−113.43 (14)
N2—C5—C6—C7	−0.2 (2)	C11—O10—C9—O13	−46.27 (16)
C3—N2—C5—C6	−175.57 (12)	C11—O10—C9—C10	75.76 (15)
C3—N2—C8A—N1	−2.82 (15)	C14—O13—C9—O10	−69.90 (14)
C3—N2—C8A—C8	176.85 (11)	C14—O13—C9—C10	163.79 (11)
C3—C16—C17—C18	179.20 (13)	C16—C3—C10—O2	−27.96 (16)
C3—C16—C21—C20	−178.63 (14)	C16—C3—C10—N1	−147.93 (11)
C5—N2—C3—C10	−166.53 (12)	C16—C3—C10—C9	95.92 (13)
C5—N2—C3—C16	−38.96 (17)	C16—C17—C18—C19	−0.7 (2)
C5—N2—C8A—N1	−178.82 (11)	C17—C16—C21—C20	−0.5 (2)
C5—N2—C8A—C8	0.85 (19)	C17—C18—C19—C20	−0.4 (3)
C5—C6—C7—C8	0.0 (2)	C18—C19—C20—C21	1.1 (3)
C6—C7—C8—C8A	0.6 (2)	C19—C20—C21—C16	−0.6 (3)
C7—C8—C8A—N1	178.60 (13)	C21—C16—C17—C18	1.1 (2)

### 6-Bromo-2-(diethoxymethyl)-2-hydroxy-3-phenyl-2,3-dihydro-1H-imidazo[1,2-a]pyridin-4-ium chloride acetonitrile monosolvate Hydrogen-bond geometry (Å, º)

**Table:**

*D*—H···*A*	*D*—H	H···*A*	*D*···*A*	*D*—H···*A*
O2—H2···Cl1	0.80 (2)	2.25 (2)	3.0489 (10)	177 (2)
N1—H1···Cl1^i^	0.86 (2)	2.37 (2)	3.1829 (12)	157.0 (17)
C7—H7···N22*A*^ii^	0.95	2.51	3.410 (15)	157
C24*A*—H24*F*···O13	0.98	2.34	3.260 (12)	156
C3—H3···Cl1^iii^	1.00	2.62	3.6125 (13)	171
C9—H9···Cl1^iii^	1.00	2.72	3.6643 (14)	157
C17—H17···Cl1	0.95	2.70	3.6259 (15)	165

Symmetry codes: (i) −*x*+1, −*y*+1, −*z*+1; (ii) *x*, *y*, *z*−1; (iii) *x*+1, *y*, *z*.
